# PA28α/β Promote Breast Cancer Cell Invasion and Metastasis via Down-Regulation of CDK15

**DOI:** 10.3389/fonc.2019.01283

**Published:** 2019-11-22

**Authors:** Shengnan Li, Xiaoqin Dai, Kunxiang Gong, Kai Song, Fang Tai, Jian Shi

**Affiliations:** ^1^Department of Pathology, School of Basic Medical Sciences, Southern Medical University, Guangdong, China; ^2^Department of Pathology, Nanfang Hospital, Southern Medical University, Guangdong, China; ^3^Guangdong Province Key Laboratory of Molecular Tumor Pathology, Southern Medical University, Guangdong, China; ^4^Department of Pathology, The First Affiliated Hospital, Sun Yat-sen University, Guangdong, China

**Keywords:** breast cancer, invasion, metastasis, PA28α/β, CDK15

## Abstract

PA28α/β activated immunoproteasome frequently participates in MHC class I antigen processing, however, whether it is involved in breast tumor progression remains largely unclear. Here, our evidences show that PA28α/β proteins are responsible for breast cancer cell migration, invasion, and metastasis. Knockdown of immunoproteasome core subunit β5i also robustly suppresses the tumor cell migration and invasion. Interestingly, silencing of PA28α/β and β5i up-regulates the protein expression of cyclin-dependent kinase 15 (CDK15). Our data further indicate that the loss of CDK15 is important for breast tumor cell invasion and metastasis. Taken together, this study implicates that targeting of PA28α/β represents a potential way for treatment of metastatic breast cancer.

## Introduction

Ubiquitin proteasome system (UPS) is critical for protein homeostasis in normal cells (1–3). Owing to a lot of genetic aberrations, there is high level of protein turnover in tumor cells, indicative of the significance of therapeutic intervention of UPS for cancer treatment (4). UPS includes three major types of proteasome, constitutive proteasome, and additional two immune-type proteasomes, thymoproteasome and immunoproteasome in higher organisms (5–8). The 20S immunoproteasome has particular cytokine-inducible subunits β1i, β2i, and β5i that are homolog to β1, β2, and β5 subunits of constitutive proteasome (9–11). These cytokine–inducible subunits facilitate immunoproteasome-generated peptide ligands of MHC class I molecules (12–15). Similar to constitutive proteasome, immunoproteasome is also involved in clearance of targeted protein (16, 17). Immunoproteasome subunit β5i specifically degrades ATRAP protein and promotes Angiotensin II-induced atrial fibrillation (18). Kovacsics et al. identified that immunoproteasome mediated degradation of heme oxygenase-1 in interferon gamma-stimulated astrocytes contributes to the neurocognitive impairment in HIV-associated neurocognitive disorders (19).

11S regulator is a basic component of immunoproteasome (17). As a critical activator of the 20S catalytic subunits, 11S undertakes essential role in the processing of downstream substrates (20). It is now clear that PA28α and PA28β proteins combine to form stable hetero-heptamers and constitute the main structure of 11S activator (21–24), which enhance the ability of immunoproteasome producing the ligands for MHC-I molecules (25). Interestingly, PA28α/β-knockout mice show decreased ability of ATP-dependent protein degradation (26). Recently, PA28α/β have been showed to associate with a various of cellular processes including tumorigenesis and development (27–30). Several studies suggested that PA28β is downregulated in gastric cancer and esophageal squamous cell carcinoma, indicative of its tumor suppressive role in these types of cancer (31, 32). Also, a series of studies have revealed the roles of β5i (LMP7) in tumor progression. Genetic deficiency or pharmacological inhibition of LMP7 blocks colon cancer initiation and progression (33). LMP-7 selective inhibition also suppresses growth and triggers apoptosis in multiple myeloma (MM) cell lines and primary patient MM cells (34). Integrative genomic and proteomic analysis of non-small cell lung carcinoma (NSCLC) cell lines revealed significantly reduced expression of immunoproteasome components. Low expression of immunoproteasome subunits in early stage NSCLC patients is associated with recurrence and metastasis (35). However, the functional roles of immunoproteasome components in breast cancer progression still remain obscure.

To identify potential drug targets of breast cancer, in this study we investigate the biological roles of PA28α/β in the proliferation, invasion, and metastasis of breast cancer cells, providing potential strategies for treatment of metastatic breast cancer.

## Results

### Silencing of PA28α/β Inhibits Breast Cancer Cell Migration and Invasion

**Figure 1:**
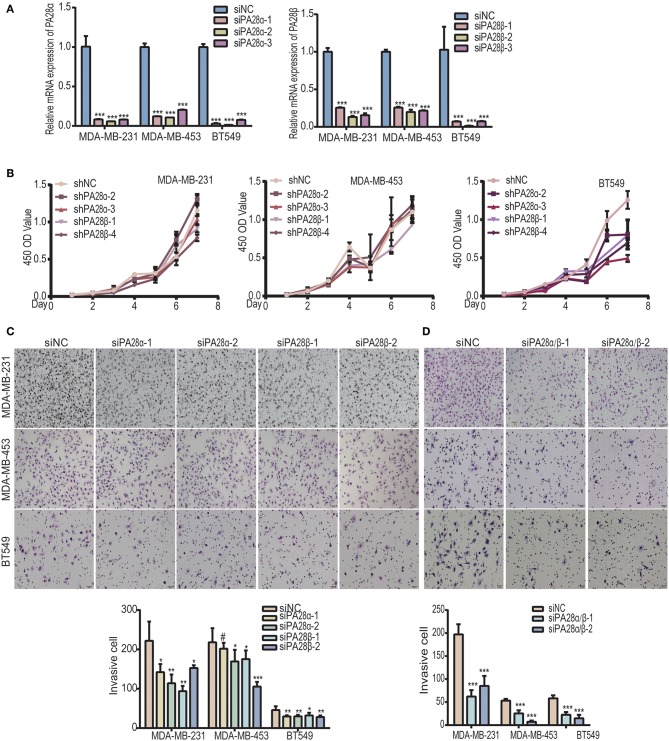
Knockdown of PA28α/β represses breast tumor cell migration and invasion. **(A)** RT-PCR assay was performed to analyze PA28α/β expression in breast cancer cell lines MDA-MB-231, MDA-MB-453, and BT549 that transfected with scramble control and siRNA (***P < 0.001). **(B)** CCK8 assay was used to detect the growth rate of PA28α/β-knockdown cells and the scramble-siRNA-control transfected cells. **(C)** Transient knockdown of PA28α or PA28β in breast cancer cells reduced invasion as revealed by Transwell assay. Representative images and quantification data are shown (NS, *P*>0.05; *P < 0.05; **P < 0.005; ***P < 0.001). **(D)** SiRNA-mediated double knockdown of PA28α/β inhibited cell invasion as detected by Transwell assay. Representative images and quantification data are shown (***P < 0.001). ^#^Indicates no significance.

PA28α/β proteins constitute 11S activator which is fundamental for the catalytic activity of immunoproteasome (20, 22). To study the functional roles of PA28α/β proteins in breast tumor progression, we knocked down PA28α and PA28β using two groups of small interfering RNAs, respectively (Figure 1A). CCK8 experiments revealed that there is no significant difference between the growth rate of PA28α/β-knockdown cells and that of control MDA-MB-231 and MDA-MB-453 cells, except for BT549 (Figure 1B). Next, we sought to detect the effect of PA28α/β-knockdown on cell motility. Our data showed that tumor cell invasive ability was greatly attenuated upon PA28α/β-knockdown in MDA-MB-231, MDA-MB-453, and BT549 cells (Figure 1C). Meanwhile, obviously slower wound closure was observed in PA28α- or PA28β-silencing breast cancer cells (Figure S1A). Moreover, double silencing of PA28α/β also significantly suppressed breast cancer cell invasion (Figure 1D). Similar results were observed from wound healing assay (Figure S1B). These data indicate that PA28α/β proteins are involved in the regulation of breast cancer cell motility.

### PA28α/β Proteins Are Responsible for Breast Cancer Cell Metastasis

**Figure 2:**
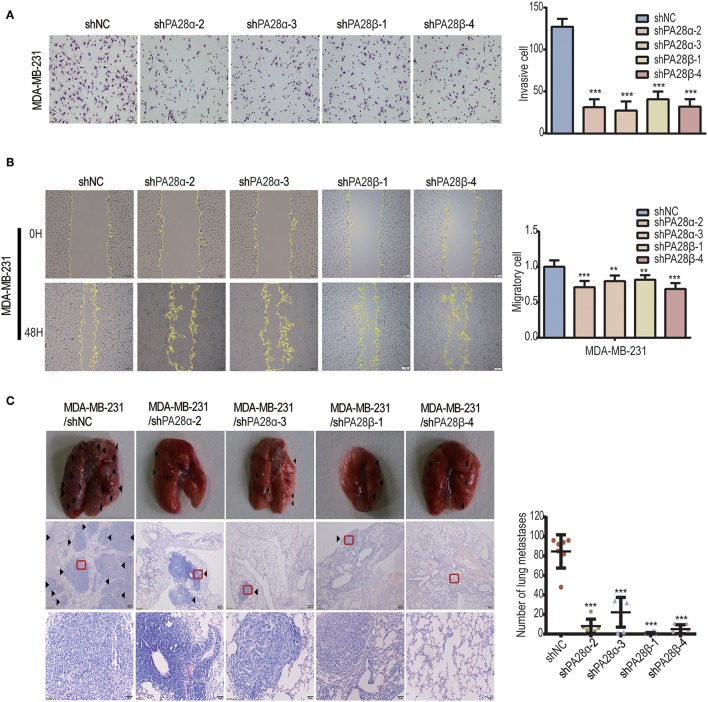
PA28α/β proteins are responsible for breast cancer cell metastasis. **(A)** Stable silencing of PA28α/β by shRNA repressed invasion of MDA-MB-231 cells as revealed by Transwell assay. Representative images and quantification data are shown (***P < 0.001). **(B)** Stable silencing of PA28α/β in MDA-MB-231 cells by shRNA suppressed migration which detected by wound healing assay. Representative images and quantification data are shown (**P < 0.005; ***P < 0.001). **(C)** Photographs of pulmonary metastasis nodules under macro-and microscope. Images of H&E staining were captured using 40× (middle) and 200× (bottom) magnitudes, respectively. Numbers of lung tumor nodules are shown (***P < 0.001).

To further study the influence of down-regulated PA28α/β levels on breast cancer cells, we constructed stable PA28α/β-silencing clones in MDA-MB-231 cells by transfection of four lentiviral plasmids carrying specific shRNA toward PA28α/β (Figure S2A). Consistently, stable knockdown of PA28α/β led to a significant decrease of cell invasion (Figure 2A) and migration (Figure 2B) compared with the vector control groups. Then, we performed tail vein injection to construct *in vivo* breast cancer lung metastasis model, measuring the metastatic capacity of vector control and PA28α/β-silencing MDA-MB-231 cells. As shown, robust reduction of pulmonary nodules from PA28α/β-knockdown clones was observed by both macroscopy and microscopy (Figure 2C; Figure S2B), which the amounts of metastatic nodules of PA28α/β-knockdown clones dropped 60~90% compared with vector control group. All these data indicate that PA28α/β proteins are required for breast cancer cell migration, invasion, and metastasis.

### Knockdown of PA28α/β Up-Regulates the Protein Expression of CDK15

**Figure 3:**
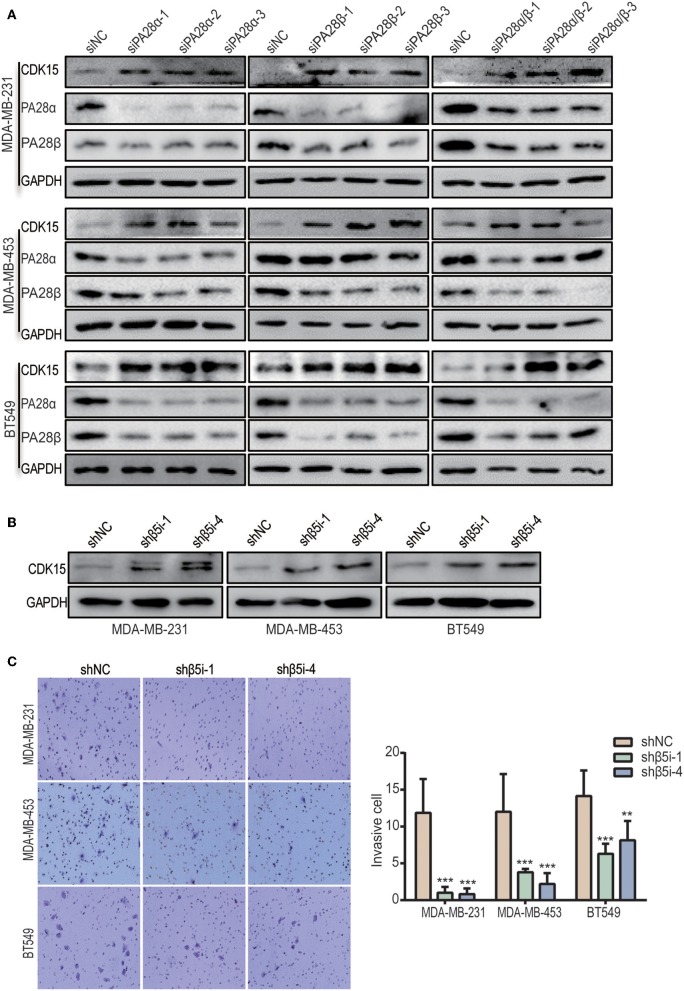
Inhibition of immunoproteasome rescues the protein expression of CDK15. **(A)** CDK15 protein levels were detected in three breast cancer cell lines when PA28α and/or PA28β was knocked down by siRNA. **(B)** CDK15 protein expression was observed in three breast cancer cell lines when β5i was stably silenced by shRNA. **(C)** Cell invasive ability was observed in vector control and β5i-knockdown breast cancer cells. Representative images and quantification data are shown (**P < 0.005; ***P < 0.001).

To look for the potential downstream proteins of PA28α/β, we detected the expression of a group of signaling molecules when PA28α/β were knocked down. Intriguingly, significantly elevated protein expression of cyclin-dependent kinase 15 (CDK15) was observed in PA28α/β-knockdown MDA-MB-231, MDA-MB-453, and BT549 cells (Figure 3A). CDK15 is a member of cyclin-dependent kinase family with unknown biological function. To investigate whether immunoproteasome is involved in regulation of endogenous CDK15 level, we generated stable knockdown clones of β5i, a core catalytic subunit of immunoproteasome (Figure S3A). As expected, CDK15 expression was also significantly up-regulated in breast cancer cells when β5i had been knocked down (Figure 3B). Similar to the outcomes of PA28α/β-silencing, loss of β5i also greatly inhibited the invasive and migratory ability of breast cancer cells (Figure 3C and Figure S3B), however, β5i-silencing did not slow down the proliferation rate (Figure S3C). These data implicate that immunoproteasome participates in the regulation of CDK15 protein level.

### PA28α/β-Induced Cell Migration and Invasion Are Partially Dependent on Down-Regulation of CDK15

**Figure 4:**
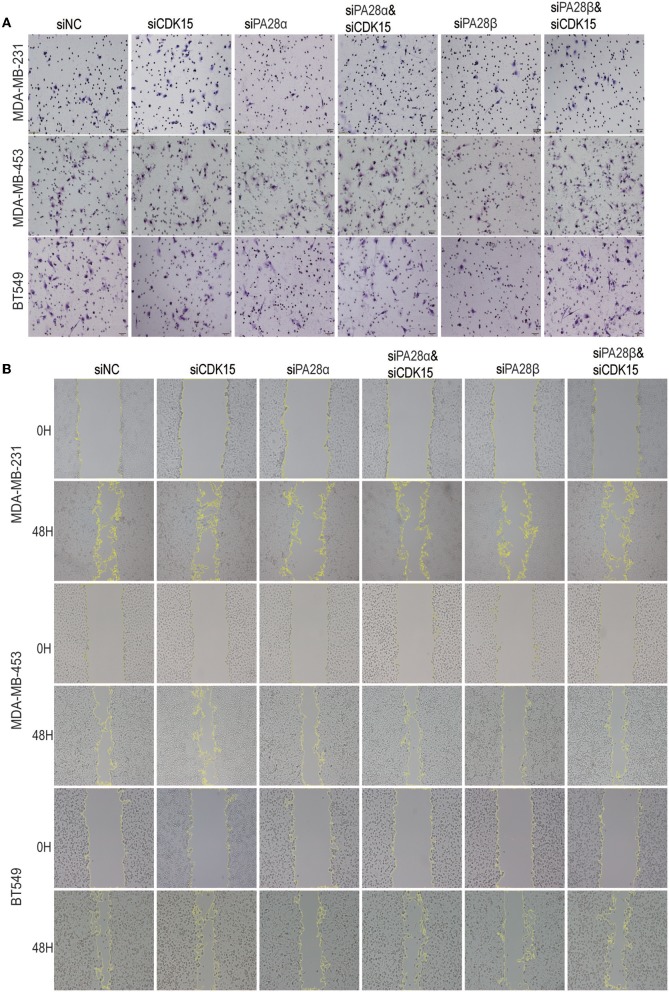
PA28α/β-induced cell migration and invasion partially depend on down-regulation of CDK15. **(A)** Breast cancer cells were singly transfected with siRNA of PA28α, PA28β and CDK15, or were co-silenced with siRNA of PA28α/CDK15 and PA28β/CDK15. Cell invasive ability was measured by Transwell assay and typical images are shown. **(B)** Breast cancer cells were singly transfected with siRNA of PA28α, PA28β and CDK15, or were co-silenced with siRNA of PA28α/CDK15 and PA28β/CDK15. Cell migration was observed by wound healing assay and typical images are shown.

We proceeded to study the functional dependence between PA28α/β and CDK15. After screen of effective siRNAs for CDK15 (Figure S4), we silenced PA28α/β and/or CDK15 in MDA-MB-231, MDA-MB-453, and BT549 cells. Owing to the low endogenous CDK15 expression in these cell lines, single knockdown of CDK15 did not influence the cell invasion, while single silencing of PA28α, or PA28β significantly repressed the invasive ability of these cells, and co-knockdown of CDK15 partially ameliorated siPA28α/β-induced effects (Figure 4A; Figure S4B). Similar results were obtained from wound healing migration assay (Figure 4B; Figure S4C). These data implicate that PA28α/β-mediated cell migration and invasion are, at least partially, dependent on regulation of CDK15.

### CDK15 Protein Is Lost in Breast Cancer

**Figure 5:**
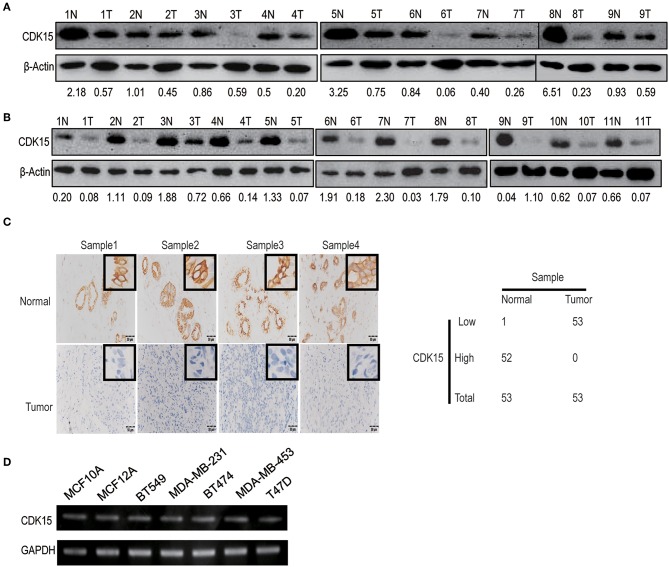
CDK15 protein is lost in breast cancer. **(A)** Western blotting assay was performed to analyze CDK15 levels in 9 pairs of non-cancerous and breast cancer tissues of luminal-subtype patients. **(B)** Western blot detection of CDK15 expression in 11 pairs of non-cancerous and breast cancer samples of basal-like patients. **(C)** Immunohistochemistry analysis of CDK15 expression from 53 pairs of breast cancer patient samples. Typical images of staining and statistical data of staining intensity are shown. **(D)** CDK15 mRNA levels were measured by PCR in a series of normal breast and cancer cell lines.

Our data implicate that decreased expression of CDK15 might be related to the regulation of cell motility. To study the relevance of CDK15 to breast cancer, we detected CDK15 protein expression in breast cancer specimens and corresponding normal tissues. Immunoblot data revealed the expression of CDK15 is frequently decreased in a range of specimens including luminal breast cancer (Figure 5A) and basal-like subtype (Figure 5B). Meanwhile, we evaluated the expression intensity of CDK15 using immunohistochemical staining in 53 pairs of breast cancer specimen and adjacent non-tumor tissue. In almost all cases, CDK15 expression in paired non-tumor tissues was positive or strongly positive, while most of tumor specimens showed negative or weak CDK15 expression (Figure 5C). We further measured the mRNA expression status of CDK15 in a group of breast cell lines, including human normal mammary epithelial cells MCF10A and MCF12A, basal-like breast cancer cells MDA-MB-231 and BT549, luminal breast cancer cells BT474, MDA-MB-453, and T47D. PCR result found that CDK15 mRNA level remains relatively constant among all of cell lines (Figure 5D), suggesting that the loss of CDK15 in breast cancer might be due to post-transcriptional regulation, which is consistent to the data that PA28α/β are responsible for down-regulation of protein level of CDK15.

### CDK15 Does Not Regulate the Tumor Cell Proliferation

**Figure 6:**
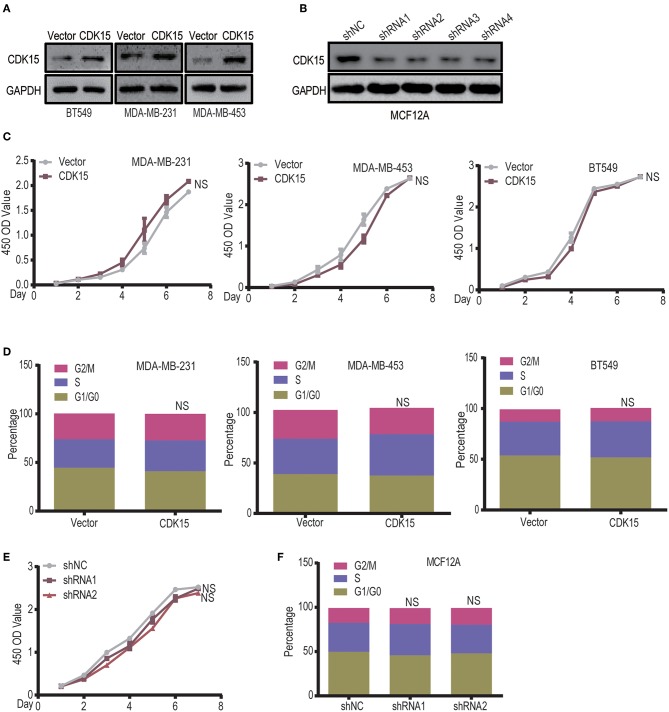
CDK15 does not regulate the tumor cell proliferation. **(A)** Expression statuses of CDK15 in three breast cancer cell lines were detected, which the cells were transfected with vector control and lenti-virus plasmid encoding CDK15. **(B)** CDK15 was knocked down in MCF12A cells by a set of shRNAs. **(C)** The effects of CDK15 over-expression on the proliferation of breast cancer cell lines were detected by CCK8 assay (NS, *P* > 0.05). **(D)** Cell-cycle analyses of breast cancer cell lines were done by flow cytometry when CDK15 was over-expressed (NS, *P* > 0.05). **(E,F)** Cell proliferation rate was detected in vector control and CDK15-knockdown MCF12A cells (*E*, CCK-8 assay; *F*, Cell cycle analyses; NS, *P* > 0.05).

To explore the significance of CDK15 deficiency in breast cancer, we established CDK15-overexpression stable clones in BT549, MDA-MB-231 and MDA-MB-453 cells that have low or undetectable levels of endogenous CDK15; on the other hand, stable CDK15-knockdown clone was constructed in MCF12A normal breast cells by shRNA-mediated silencing (Figures 6A,B). Due to the nature of cyclin-dependent kinase, we first investigated the effect of CDK15 on cell proliferation. However, overexpression of CDK15 showed only slight or no effect on tumor cell growth (Figure 6C). In addition, the cell cycle distribution did not alter significantly in CDK15-overexpressing tumor cells compared with their vector control cells (Figure 6D). Consistently, knockdown of CDK15 in MCF12A cells also did not affect the growth potential and cell cycle distribution (Figures 6E,F). These results reveal that CDK15 protein is not involved in regulation of cell proliferation and cell-cycle progression.

### CDK15 Negatively Modulates Breast Cancer Cell Invasion and Metastasis

**Figure 7:**
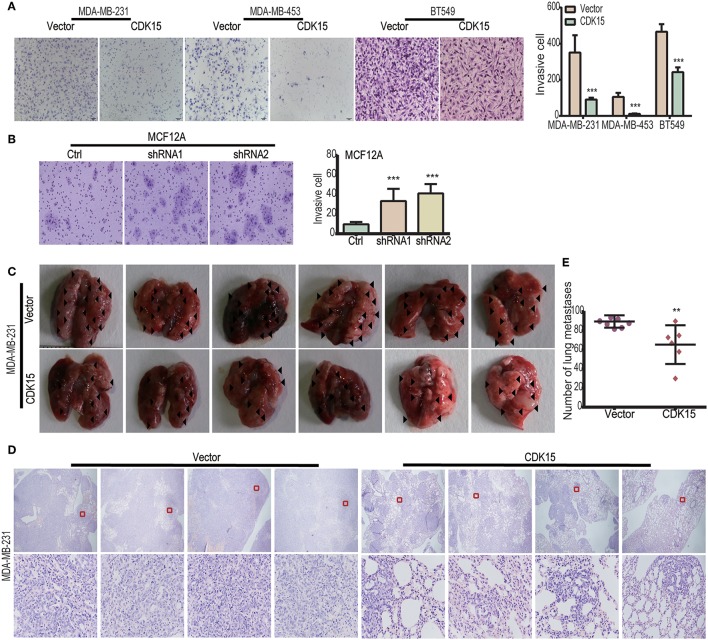
CDK15 negatively modulates breast cancer cell invasion and metastasis. **(A)** Transwell assay was performed in vector control and CDK15 overexpressing MDA-MB-231, BT549, and MDA-MB-453 cells. Representative images and quantification data are shown (***P < 0.001). **(B)** Cell invasive ability was measured in vector control and CDK15-knockdown MCF12A cells. Representative images and quantification data are shown (***P < 0.001). **(C)** Photographs of lung macro-metastases from vector control and CDK15-overexpressing mice groups are shown. **(D)** Hematoxylin and eosin (H&E) staining of lung sections was performed. Images were captured by 40× (*top*) and 200× (*bottom*) magnitudes, respectively. **(E)** Quantification of mice lung tumor nodules from vector control and CDK15-overexpressing groups is shown (**P < 0.005).

Although CDK15 does not regulate proliferative potential of breast cancer cells, intriguingly, CDK15-overexpressing cells lost their mesenchymal phenotype and acquired epithelial morphology (Figure S5A); conversely, CDK15-silencing MCF12A cells obtained obvious spindle-like morphology compared with vector control cells (Figure S5B), suggesting that CDK15 might be related to the regulation of cell motility. Then we tried to investigate the function of CDK15 in cell migration and invasion processes. Our results found that the cell number of vector control group that passing through the matrigel was 3-fold higher than the CDK15-overexpression group in MDA-MB-231, 5-fold higher in MDA-MB-453 and 2-fold higher in BT549, respectively (Figure 7A). In addition, analyses of wound healing in various groups indicated that the CDK15-overexpressing tumor cells had significantly weaker migratory ability than vector control cells (Figure S5C). Consistently, knockdown of CDK15 in MCF12A enhanced its ability of invasion (Figure 7B) and migration (Figure S5D). To study its role *in vivo*, the pulmonary metastasis model was established in nude mice which injected with vector control and CDK15-overexpressing MDA-MB-231 cells via tail vein injection. Four weeks after injection, mice were sacrificed, and pulmonary metastasis nodules were counted under macro-and microscope. More metastasis nodules were found in the vector control group mice, while CDK15-overexpressing group exhibited much less nodules (Figures 7C–E). These data suggest that CDK15 functions as a suppressor of breast cancer cell invasion and metastasis.

## Discussion

Our study uncovers the pro-invasive and pro-metastatic roles of PA28α/β. Several studies have indicated that ubiquitin proteasome system dysregulation favors the development of many types of cancer. UPS inhibition has become an important strategy for drug development in cancer treatment (36–38). Here, our data showed that silencing of PA28α/β robustly suppresses breast tumor cell migration and invasion *in vitro* as well as metastatic ability *in vivo*; similarly, knockdown of immunoproteasome core subunit β5i also greatly represses the invasion of breast cancer cells. Interestingly, silencing of PA28α/β or β5i did not affect the growth rate of tumor cells. These observations suggest that specific pharmacological targeting of immunoproteasome is probably effective for the treatment of metastatic breast cancer.

Our study also reveals CDK15 as a suppressor of breast cancer cell invasion and metastasis. Cyclin-dependent kinases (CDKs) family includes a series of serine/threonine protein kinases, which usually function as oncogene and have been found to play critical roles in cell cycle progression by co-operating with multiple cyclins (39–41). Recently, small inhibitors of CDK4/6 have been used for restraint of tumor progression through induction of cell cycle arrest (42). Additionally, some CDK family members are involved in regulation of gene transcription. For example, CDK9 mediates transcriptional activation via regulation of RNA polymerase II activity (43). In the present study, we focused on CDK15, a CDK family member with unknown function. *CDK15* gene maps to chromosome 2q33.1 and has an important paralog gene, *CDK14*. A recent research found that Epstein-Barr virus (EBV) integration into the introns can decrease the expression of *CDK15* gene in nasopharyngeal carcinomas, implicating that downregulation of CDK15 may contribute to tumor development (44). Another study reported that CDK15 attenuates Tumor necrosis factor-related apoptosis-inducing ligand (TRAIL) induced apoptosis by inducing phosphorylation of Survivin (45). Here, our analyses in breast tumor specimens and breast cancer cell lines indicated that CDK15 protein level is down-regulated in breast cancer, while its mRNA expression keeps constant. CCK8 and cell cycle analyses confirmed that alteration of intrinsic CDK15 level does not influence the tumor cell proliferation potential. Intriguingly, we found that CDK15 negatively modulates the cancer cell motility, as evidenced by the facts that overexpression of CDK15 in breast cancer cells significantly repressed their migration, invasion and metastasis; while knockdown of CDK15 in normal breast cells greatly enhanced the migratory and invasive capacity. However, the underlying mechanism of CDK15 in the regulation of cell motility still remains obscure, we will proceed to discover its downstream phosphorylation substrates or interacting partners, for example, Cyclin proteins. Interestingly, although CDK14 shares similar amino acid sequence with CDK15, the functions of these two PFTK family members are different from each other. CDK14 has been reported to act as a Cyclin Y-dependent protein kinase and promote tumor progression. Especially, CDK14 is positively associated with high-motility phenotype of tumor cells. In the future, we will try to clarify the molecular basis of the functional difference between CDK15 and CDK14.

The study implicates the working mechanism of PA28α/β mediated tumor cell invasion and metastasis, also the post-translational regulation of CDK15 protein. We found that knockdown of PA28α/β rescues the protein expression of CDK15 and co-silencing of CDK15 is able to ameliorate the suppressive role mediated by knockdown of PA28α/β on cell migration and invasion, suggesting that CDK15 might be a potential downstream target of 11S regulator and immunoproteasome. However, we failed to identify the interaction between CDK15 and β5i or 11S regulator, therefore, we are not certain whether CDK15 is the direct protein substrate of 11S and immunoproteasome. Meanwhile, downregulation of CDK15 may be not the unique working mechanism of 11S in the facilitation of tumor cell motility, we cannot exclude the possibility that other downstream proteins are involved in the pro-invasive and pro-metastatic function of PA28α/β. Our follow-up study will focus on whether immunoproteasome is responsible for direct degradation of CDK15 and search of other downstream target proteins of immunoproteasome in tumor cells.

## Materials and Methods

### Cell Culture and Patient Samples

Breast cancer cell lines BT549 and T47D were maintained in RPMI-1640 medium with 10% fetal bovine serum. Breast Cancer cell lines MDA-MB-231, MDA-MB-453, MCF7, BT474, and human mammary epithelial cells MCF12A were cultured using Dulbecco's Modified Eagle Medium containing 10% FBS. Penicillin/streptomycin were added to culture medium. Stable cell clones were constructed by selection using puromycin (1 μg/mL). The fresh surgically resected breast cancer tissues and adjacent normal tissues were obtained from Guangdong General Hospital (Guangzhou, China).

### Hematoxylin-Eosin (H and E) and Immunohistochemical Staining

Specimens were fixed in 10% paraformaldehyde overnight, embedded in paraffin, serially sectioned (2.5 μM) and stained with hematoxylin eosin. Tissue sections were baked at 68°C for 2 h. Tissue sections were deparaffinized with 3 washes of xylene, followed by rehydration with successive washes of ethanol 100, 95, 90, 80, 70%. Samples then were stained with hematoxylin and eosin, successively dehydrated with washes of gradient alcohol and xylene dehydration, dried and mount before imaging. For IHC, blocked endogenous peroxidase by incubation in 3% H_2_O_2_ for 30 min and then washed three times per wash for 5 min in PBS. Sections were incubated with primary antibody at a dilution above overnight at −4°C, followed by incubation with rabbit antibody for 30 min at 37 °C. The enzyme was visualized after 3-min incubation with diaminobenzidine (DAB). Primary antibodies specific for CDK15 (GeneTex 1:50), PA28α (Cell signaling 1:100), PA28β (Cell signaling 1:100) were used for immunohistochemistry.

### Virus Production, Cell Transduction, and siRNA Transfection

HEK293T cells were seeded at 5 × 10^5^ cells per 60 mm dish and cultivated for 12–16 h. Lentivirus were produced by co-transfection of expression vectors and packaging plasmids psPAX2 and pMD2.G in a ratio of 4:3:1. The supernatant was collected 48 h after transfection and filtered through a 0.45 μM membrane filter, then stored at −80°C until be used. For infection, 1 × 10^5^ cells were plated in the 6-well plates. After 16 h, the filtered supernatant was added to each plate that treated with 8 μg/ml polybrene. Stable clones were selected with 1 μg/ml puromycin. For siRNA transfection, 1 × 10^5^ cells were plated into 6-well plates and transfected with siRNA using siRNA-mate reagent (GenePharma) after reaching 70% confluency.

### Western Blotting

Cells were harvested and lysed, and protein concentrations were determined by BCA protein assay kit. Equal amounts of purified proteins were separated by 10% SDS-PAGE and transferred onto PVDF membranes. Western signals were detected using Enhanced Chemiluminescence kit (FDbio science). Primary antibodies were used as follows: CDK15 (1:300, GeneTex), PA28α (1:1,000, Cell signaling), PA28β (1:700, Cell signaling), GAPDH (1:1,000, Proteintech), β-Actin (1:1,000, Transkgen). Anti–rabbit or anti-mouse secondary antibody (Earthox) was used at 1:5,000 dilution.

### RT-PCR Analysis

Total RNA was extracted using Trizol extraction (Invitrogen). Then 1 μg of RNA was reverse transcribed using SuperScript III kit following manufacturer instruction. Values of sample relative to control were calculated using the ΔΔCT method. *GAPDH* was used as a housekeeping gene for normalization. Results were represented as fold change. The sequences of primers used for qPCR analysis were listed as follows:

GAPDH Forward GGAGTCCACTGGCGTCTT
GAPDH Reverse TCTTGAGGCTGTTGTCATACTT
CDK15 Forward ATGTCTCAGCATCCAGGAGG
CDK15 Reverse CCCTGTGAAGAACGTGTTGG
PSME1 Forward CCAGTGCCTGATCCAGTCAAG
PSME1 Reverse ACCACGATCTTTTCATTGCAGT
PSME2 Forward GCAAGAGGACTCCCTCAATGT
PSME2 Reverse CTTCTGGCTTAACCAGGGCA.

### Cell Cycle Analysis

Cells were collected and fixed with 70% ethanol overnight at −20°C. Before propidium iodide (PI) staining, cells were washed twice with cold PBS, followed by resuspension with PI (KeyGEN, KGA512) staining buffer for 30 min at 37°C. 10,000 cells were acquired and analyzed with a BD flow cytometer (BD Biosciences). All data were analyzed using FlowJo (TreeStar).

### Cell Proliferation Assay

Cells were plated in triplicate at a density of 1,000 cells per well in 96-well plate. At *t* = 0, 1, 2, 3, 4, 5 days post-seeding. Cell Counting Kit-8 (CCK8) (Bimake, B34302) was incubated for 2 h at 37°C in a ratio of 1:10 in medium. Samples were measured with Bioplate Synergy 5 microplate reader at 450 nm.

### Wound-Healing and Transwell Assay

Cells were seeded at a confluent density and cultured overnight. The plate was scratched with a 200-μl pipette tip and the wound was allowed to heal for 48 h. The reduction of area between two wound edges was calculated and quantitated. For transwell assay, each insert (8-μm pore size, Falcon) was coated with matrigel overnight. The upper insert contained 1 × 10^5^ cells in 150 μl serum-free medium, and the lower chamber was filled with 600 μl complete medium as the chemo attractant. Cells were allowed to migrate for 20 h, followed by 4% formaldehyde solution fixation and haematoxylin staining. Un-migrated cells on the membrane apical side were removed using wet cotton swabs and migrated cells were counted under microscope.

### Mice Model

Balb/C female nude mice (4–6 weeks) were purchased from Animal Center of Guangdong Province. All animal experiments were approved by the Animal Care and Use Committee of Southern Medical University. Animals were randomly assigned to groups. The 2 × 10^5^ MDA-MB-231 cells were re-suspended in 0.1 ml PBS and injected into the tail vein. After 6 weeks, the mice were sacrificed, and detectable tumor nodules on the surface of whole lung were counted and quantified.

### Statistical Analysis

All statistical analyses were carried out using GraphPad Prism 6 software. Statistical significance is reported in the figures and figure Legends. Data were considered to be statistically significant when *P* < 0.05 which calculated by two-tailed *t*-test (^#^*P* > 0.05; ^*^P < 0.05; ^**^P < 0.005; ^***^P < 0.001).

## Data Availability Statement

All datasets generated for this study are included in the article/supplementary material.

## Ethics Statement

The studies involving human participants were reviewed and approved by Guangdong General Hospital. The patients/participants provided their written informed consent to participate in this study. The animal study was reviewed and approved by Animal Care and Use Committee of Southern Medical University.

## Author Contributions

JS conceived and supervised the study, designed experiments, and wrote the manuscript. SL, XD, KG, KS, and FT performed experiments and analyzed data.

### Conflict of Interest

The authors declare that the research was conducted in the absence of any commercial or financial relationships that could be construed as a potential conflict of interest.

## Supplementary Material

Figure S1 Knockdown of PA28α/β represses breast tumor cell migration and invasion. **(A)** Cell migration statuses in PA28α or PA28β-silencing breast cancer cells were observed by wound healing assay. Representative images and quantification data are shown (^*^*P* < 0.05; ^**^*P* < 0.005; ^***^*P* < 0.001). **(B)** Cell migration was detected in MDA-MB-231, MDA-MB-453, and BT549 with double silencing of PA28α/β. Representative images and quantification data are shown (^*^*P* < 0.05; ^**^*P* < 0.005; ^***^*P* < 0.001).

Figure S2 **(A)** PA28α/β proteins are responsible for breast cancer cell metastasis. RT-PCR assay was done to analyze of PA28α/β expression in control (shNC) and knockdown clones of MDA-MB-231 (NS, *P* > 0.05; ^**^*P* < 0.005; ^***^*P* < 0.001). **(B)** Photographs of lung macro-metastases from control, PA28α- and PA28β-knockdown groups are shown, black arrows indicate metastatic nodules.

Figure S3 Inhibition of PA28α/β rescues the protein expression of CDK15. **(A)** RT-PCR assay was performed to detect β5i expression in breast cancer cells that transfected with control and β5i shRNA (^***^*P* < 0.001). **(B)** Cell migration was determined in vector control and β5i-knockdown breast cancer cells. Representative images and quantification data are shown (^*^*P* < 0.05; ^**^*P* < 0.005; ^***^*P* < 0.001). **(C)** The effects of β5i-knockdown on the proliferation of breast cancer cells were detected by CCK8 assay.

Figure S4 PA28α/β-induced cell migration and invasion is partially dependent on down-regulation of CDK15. **(A)** RT-PCR experiments were performed to observe CDK15 expression in breast cancer cells that transfected with scramble control or siRNA for CDK15 (^**^*P* < 0.005; ^***^*P* < 0.001). **(B)** Breast cancer cells were singly transfected with siRNA of PA28α, PA28β and CDK15, or were co-silenced with siRNA of PA28α/CDK15 and PA28β/CDK15. Cell invasive ability was measured by Transwell assay and quantification data are shown (^*^*P* < 0.05; ^**^*P* < 0.005). **(C)** Breast cancer cells were singly transfected with siRNA of PA28α, PA28β and CDK15, or were co-silenced with siRNA of PA28α/CDK15 and PA28β/CDK15. Cell migration was observed by wound healing assay and quantification data are shown (^*^*P* < 0.05; ^**^*P* < 0.005).

Figure S5 CDK15 negatively modulates breast cancer cell invasion and metastasis. **(A)** The morphological change of CDK15 over-expressing breast cancer cells compared with vector control cells was observed by microscopy (*upper*, 100×; *bottom*, 400×). **(B)** The morphological change of CDK15-silencing MCF12A cells compared with vector control cells was observed by microscopy (*upper*, 100×; *bottom*, 400×). **(C)** Microphotograph of MDA-MB-231, BT549, and MDA-MB-453 cells immediately and 48 h after wound creation, and the wound area was quantified using Image J software (^**^*P* < 0.005; ^***^*P* < 0.001). **(D)** Microphotograph of MCF12A cells immediately and 48 h after wound creation, and the wound area was quantified using Image J software (^**^*P* < 0.005; ^***^*P* < 0.001).
