# Supplementary figures and images for: PA28α/β Promote Breast Cancer Cell Invasion and Metastasis via Down-Regulation of CDK15
Source: Front Oncol. 2019 Nov 22;9:1283. doi: 10.3389/fonc.2019.01283 (PMC6883405; doi:10.3389/fonc.2019.01283)

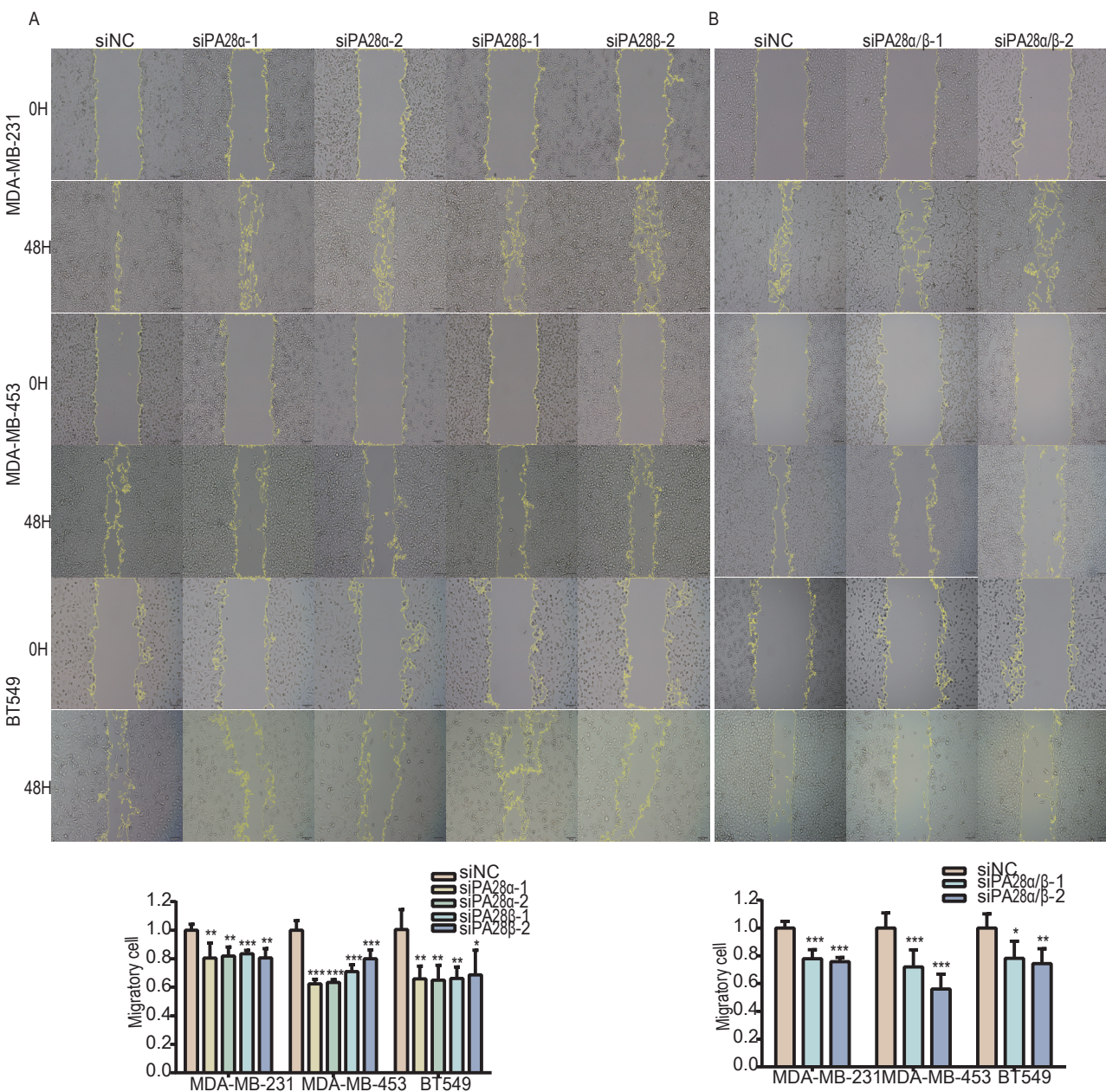

Figure S1

A

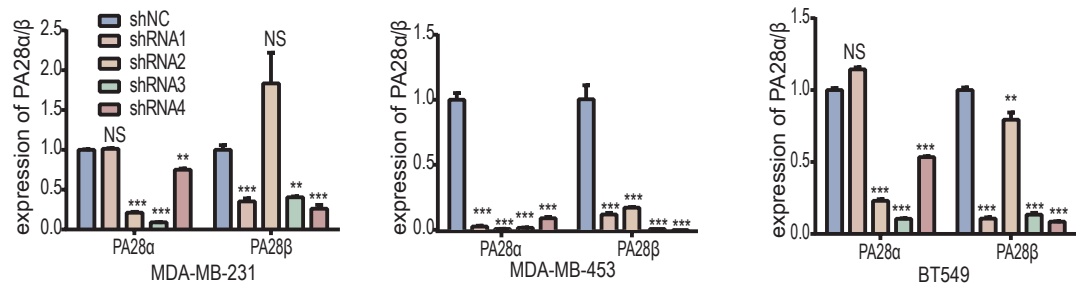

B

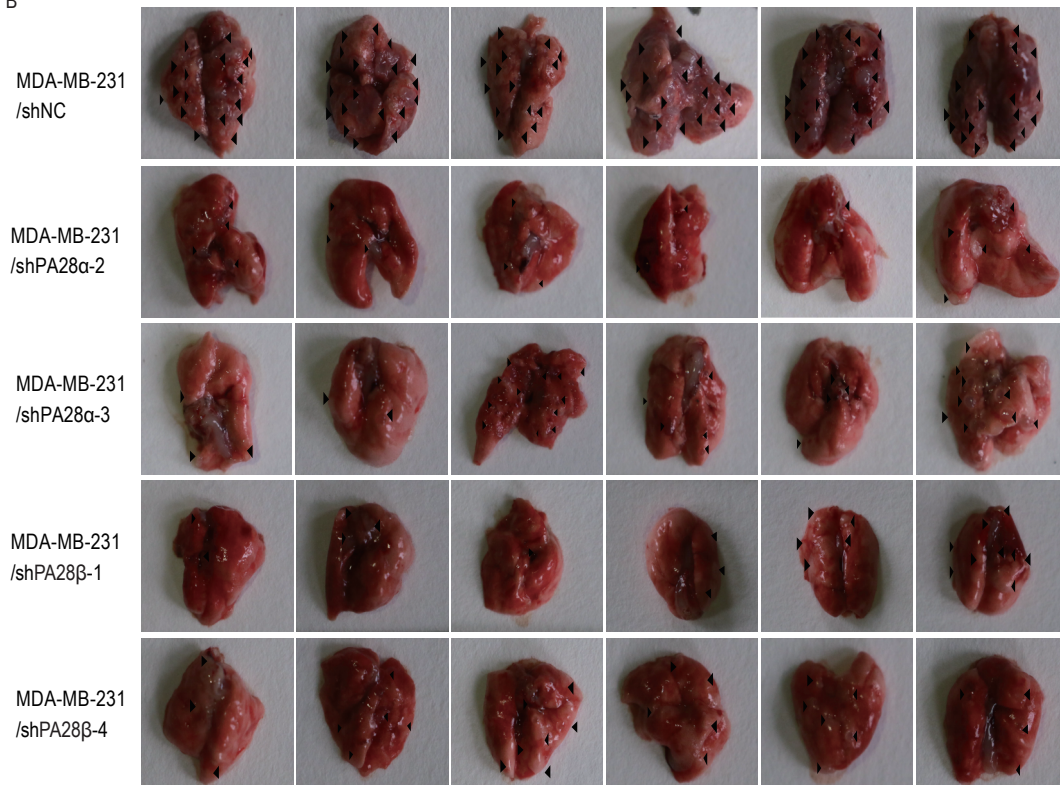

Figure S2

A

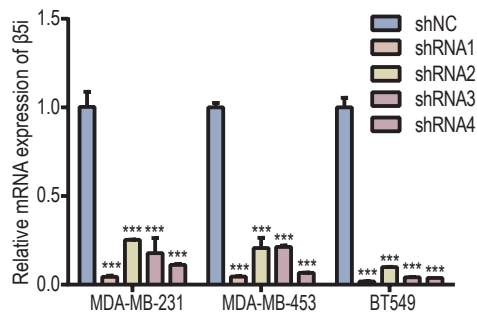

B

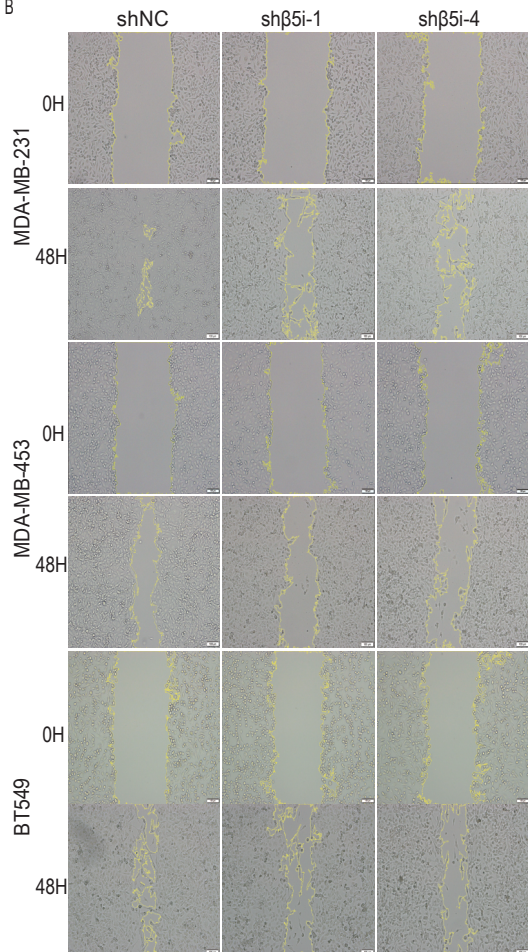

C

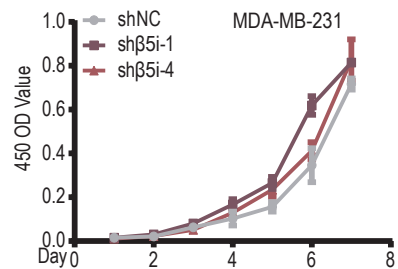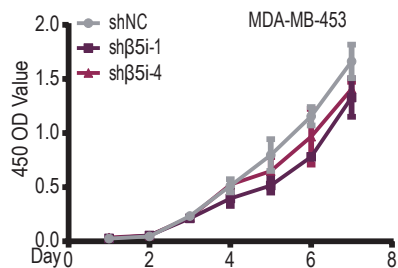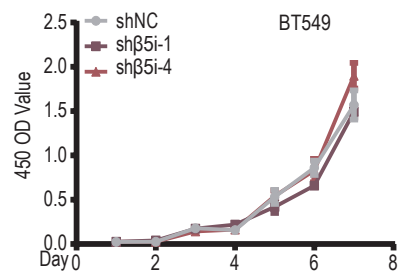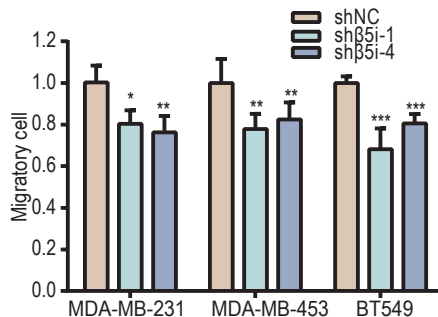

Figure S3

A

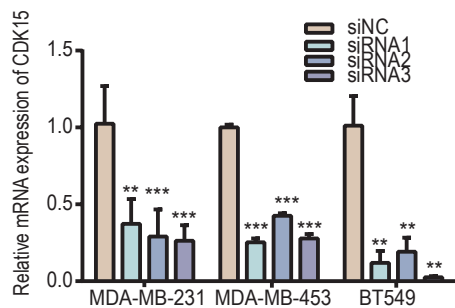

B

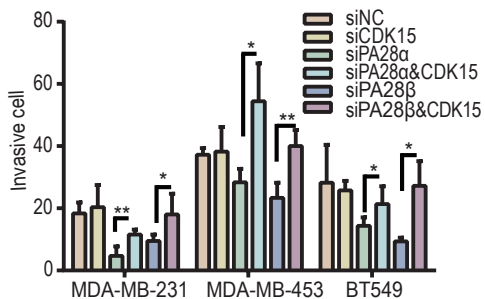

C

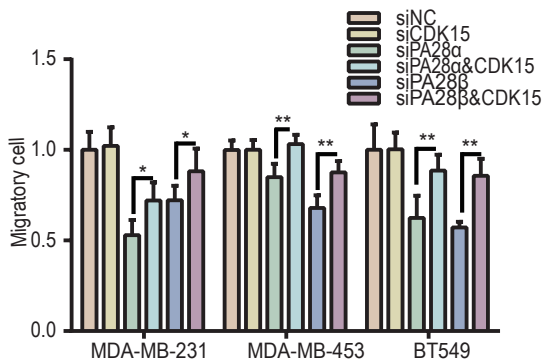

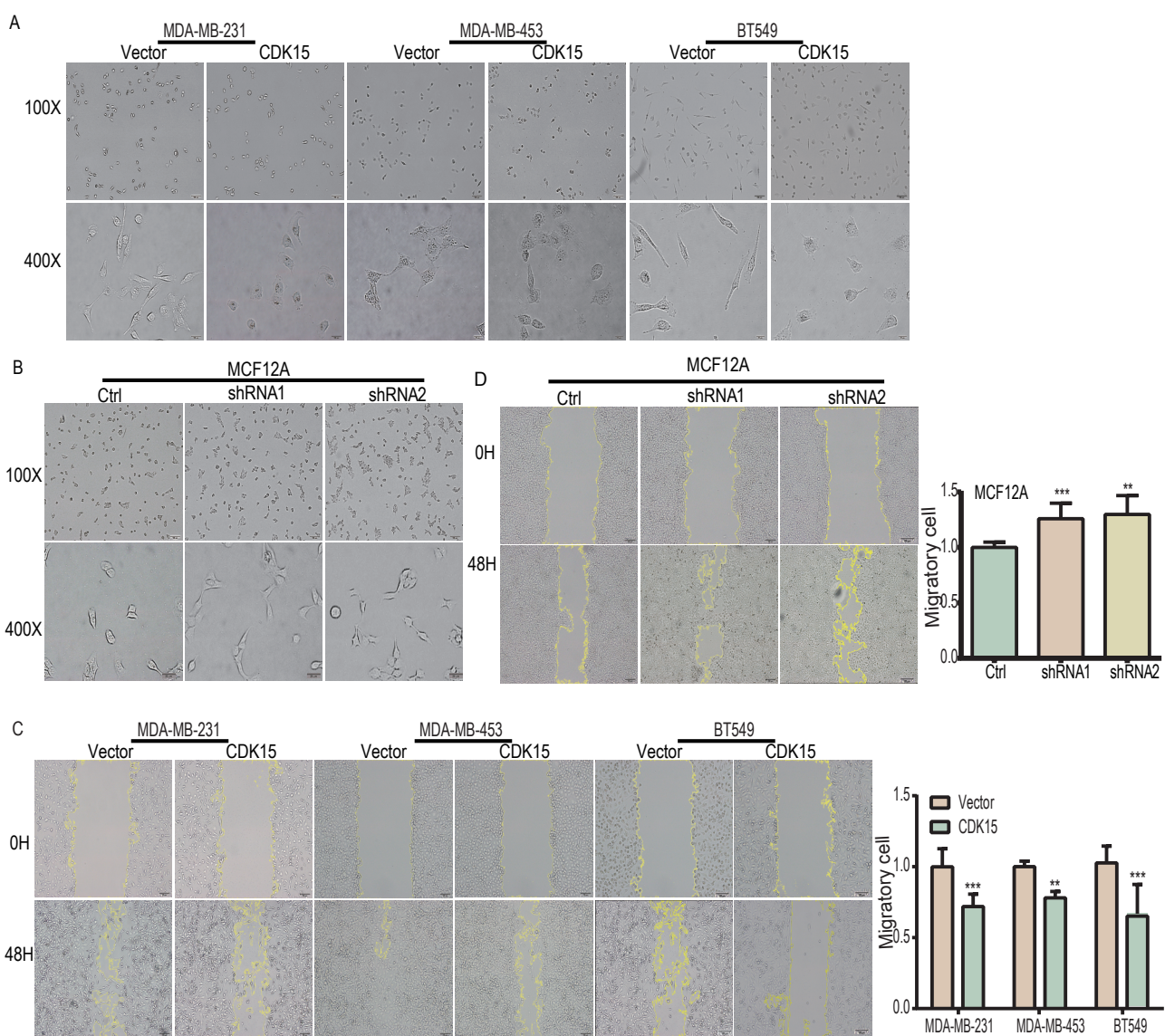

Figure S5

Supplement: Figure S1 — Knockdown of PA28α/β represses breast tumor cell migration and invasion. (A) Cell migration statuses in PA28α or PA28β-silencing breast cancer cells were observed by wound healing assay. Representative images and quantification data are shown (*P < 0.05; **P < 0.005; ***P < 0.001). (B) Cell migration was detected in MDA-MB-231, MDA-MB-453, and BT549 with double silencing of PA28α/β. Representative images and quantification data are shown (*P < 0.05; **P < 0.005; ***P < 0.001). [file Presentation_1.pdf]
